# Application of 3D navigation for osteotomy of DDH in children: A systematic review and meta-analysis

**DOI:** 10.3389/fped.2022.1021981

**Published:** 2022-11-10

**Authors:** Yunlong Liu, Yancai Yang, Sheng Ding

**Affiliations:** Department of Pediatric Surgery, Ningbo Women and Children's Hospital, Ningbo, China

**Keywords:** child DDH, hip osteotomy, 3D navigation, auxiliary, meta-analysis

## Abstract

**Objective:**

To systematically review the current articles to compare the efficacy and safety of 3D navigation-assisted osteotomy of DDH with conventional osteotomy of DDH in children. Study design Databases such as PubMed, Embase, Cochrane Library were searched, from inception to April, 2022, for studies applying 3D navigation-assisted osteotomy in DDH children.

**Methods:**

There were 626 articles identified. According to the search strategy and inclusion criteria, 7 studies were finally included, with a total of 288 cases. Study screening, data extraction, and quality assessment were conducted by two reviewers independently. Data analyses were performed using RevMan 5.4 software.

**Results:**

There were 7 retrospective cohort studies included. Meta-analysis showed that 3D navigation-assisted DDH osteotomy resulted in shorter duration of surgery [*I*^2^*^ ^*= 88%, REM, MD = 22.86, 95%CI (−27.29, −18.43), *p *< 0.00001], less radiation exposure during surgery [*I*^2^*^ ^*= 53%, REM, MD = 2.76, 95%CI (−3.15, −2.37), *p *< 0.00001], and less intraoperative bleeding [*I*^2^*^ ^*= 94%, REM, MD = 26.83, 95%CI (−39.24, −14.41), *p *< 0.0001], compared with conventional DDH osteotomy. There was a significant difference in the number of patients with McKay clinical function graded as poor between the two groups [*I*^2^*^ ^*= 0%, FEM, RR = 0.20, 95%CI (0.05, 0.74), *p *= 0.02], whereas there were no significantly statistical differences in the corrected acetabular index angle, postoperative leg length discrepancy, and number of patients with Severin x-ray graded as poor between the two groups (*p *> 0.05).

**Conclusion:**

3D navigation-assisted pelvis and thighbone osteotomy for DDH children could shorten duration of surgery and reduce intraoperative bleeding and x-ray exposure, presenting definite therapeutic effect.

**Systematic Review Registration:**

https://www.crd.york.ac.uk/PROSPERO/#myprospero, identifier: CRD42022333767.

## Introduction

Developmental dysplasia of the hip (DDH) is the most common type of hip joint deformity in children, with the morbidity of 4–6 per thousand in newborns in developed countries (1, 2). It presents a tough-nut-to-crack in child osteology especially for children aged over 2 years (3). Pelvic osteotomy combined with proximal femur varus, rotation, and shortening is the most commonly applied surgical option for DDH. However, DDH children often have aberrant and complicated anatomical characteristics in their hip joints, which poses a great challenge for surgeons to perform precise osteotomy. Pediatric orthopedists perform osteotomy in pelvic and proximal femur conventionally based on their experience. Any error in preoperative plan-making or intraoperative manipulation would compromise the outcome of the surgery, making it an urgent need for a more precise surgical approach.

3D navigation template technology uses CT data and 3D reconstruction technology to design a rotating and moving orthotic limit navigation template for hip osteotomy, which can achieve accurate osteotomy and accurate displacement of bone blocks. Three-dimensional navigation technology (3D navigation) can help clinicians make precise and individualized surgical scheme and perform surgery simulation so as to facilitate precise osteotomy and internal fixation (4). It has been widely applied in multiple fields such as complicated pelvic, hip, and spinal surgery, bone tumor resection, limb deformity orthotics, and artificial limb-manufacturing (4). However, there are few clinical studies focusing on 3D navigation-assisted hip osteotomy for children with DDH so that evidence of its efficacy and safety remains insufficient, and there is still lack of relevant systematic review and meta-analysis. Therefore, it is of great necessity to conduct a meta-analysis to evaluate the clinical efficacy of 3D navigation-assisted osteotomy for the treatment of DDH in children, so as to provide reference for clinical decision-making.

We aim to systematically review the current articles to compare the efficacy and safety of 3D navigation-assisted osteotomy of DDH with conventional osteotomy of DDH in children.

## Materials and methods

The protocol for this systematic review was established in advance, documented, and submitted before commencement to PROSPERO (registration number CRD42022333767) on June 15, 2022. This systematic review conformed to the principles detailed in the handbook of the Cochrane Collaboration and the guidelines established by the Preferred Reporting Items for Systematic Reviews and Meta analysis.

### Search strategy and study selection

PubMed, Cochrane Library, and Embase were searched from inception to April 20th, 2022. Other sources, including teaching materials, conference records, national health guidelines, systematic reviews and meta-analyses, clinical trial registrations, and DDH treatment guidelines, were manually searched. We consulted experts in literature search to search for studies that were unidentified in preliminary search. No language restriction was set. Search strategy mainly included: (Developmental Dysplasia of the Hip) AND (3d printing* OR Computer-Aided Design).

Two reviewers (YY and DS) independently browsed the titles and abstracts to identify potential eligible articles, and the full-texts of which were retrieved and read to identify eligible studies to be included. Any disagreement was settled *via* discussion or, if necessary, by a third reviewer (LY). Consensus was reached after discussion and consultation. A PRISMA flowchart was used to present the overview of study selection process.

### Inclusion and exclusion criteria

#### Inclusion criteria

(1) Types of study: Observational cohort study regarding osteotomy of DDH in children;
(2) Types of participants: Children receiving hip osteotomy who were recruited in the study after appraisal of the ethics committee and signed informed consent;
(3) Types of intervention: 3D navigation-assisted osteotomy and performing conventional osteotomy for DDH children.

#### Exclusion criteria

(1) Complications undescribed and diagnosis unclear or unclassified;
(2) Data incomplete or unavailable;
(3) Animal study, case-report, systematic review and meta-analysis, etc.;
(4) Repeated publication, uncomplete study, or study considered to be pointless.

### Data extraction

Characteristics of included studies were extracted by two reviewers (YY and DS) independently, which included: name of the first author, journal, publication date, DDH classification, number of hip joint cases in cohort, 3D navigation technology, pelvic and femur osteotomy, mean age and gender distribution of participants, and follow-up. We also extracted the radiographic results, including femur osteotomy angle, postoperative acetabular index, and leg length discrepancy. Clinical outcomes included the postoperative function score (number of reports and detailed description), intraoperative bleeding, duration of surgery, radiation exposure, and postoperative complication. Results of data extraction of the two reviewers were cross-checked to ensure the accuracy. Any discrepancy in recorded values was verified by both the two reviewers to make a final decision. For missing or incomplete data, the reviewers would try to contact the author to obtain the original data. Had they failed to contact the author, the missing or incomplete data would have been removed.

### Quality assessment

Quality assessment and data extraction of included studies were conducted by two reviewers independently. Quality assessment for retrospective cohort study was based on the Newcastle-Ottawa Scale (NOS). Any disagreement was settled through discussion with the third reviewer. All included studies were assessed in strict accordance with the assessment criteria of Cochrane Handbook 5.1.1.

### Statistical analysis

Consistency between the reviewers was quantified using *Kappa* statistics, and risk ratio (RR) was applied as pooled statistic for therapeutic effect. Statistical homogeneity was assessed using *q* test with the significance level of 10%. Heterogeneity test was performed using Higgins *I*^2^ statistic. Meta-analysis was performed *via* RevMan 5.4 software. A fixed-effect model would be applied if no significant heterogeneity was considered among the studies (*I*^2^* *< 50%), otherwise, a random-effect model would be used. However, we provided extra data fixed- and random-effect models for the groups analyzed to process data comparison. Sensitivity analysis was employed to determine the influence of each individual study on the summary results by repeating random-effects meta-analysis, omitting one study at a time. For outcome measures that could not be quantitatively pooled, a qualitative description would be provided. Publication bias assessment inapplicable in that less than 10 studies were included.

## Results

### Study selection

**Figure 1:**
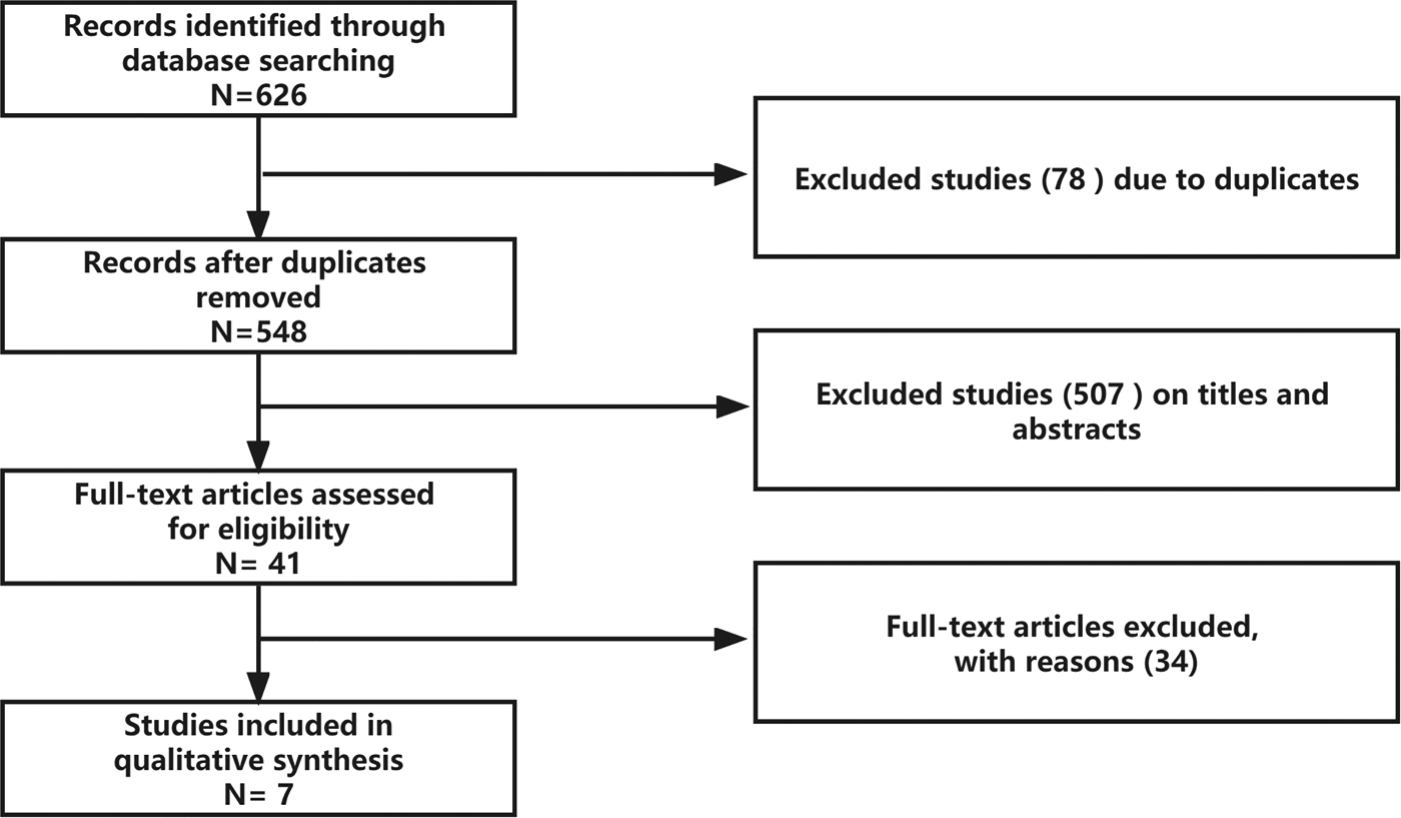
Flow diagram.

There were 626 articles retrieved, and each article was evaluated independently. There were 548 articles remained after removing 78 duplicates, then 507 relevant articles were excluded after browsing titles and abstracts (animal studies, pharmacological studies, letters, etc.), and 41 remained for full-text reading. Following exclusion of 34 studies failing to meet the inclusion criteria, 7 retrospective cohort studies were finally included in this meta-analysis (Figure 1).

### Characteristics of included studies and quality assessment

Publication date of included studies ranged from 1978 to 2022, with a total of 288 child participants. All included studies were retrospective cohort design, with similar definition of DDH but different disease classification. Among the 7 studies (5–11), 1 reported the preoperative scheme-making and clinical effect of computer-aided design (CAD) and 3D reconstruction, 2 reported the application and prospect of 3D printing in osteotomy of child DDH, 4 reported the application of 3D technology and navigation template in osteotomy of child DDH (Table 1). All groups of included studies were comparable.

**Table 1:** Characteristics of the included studies.

Author	Year	Region	Population	Age (mean, SD)	Gender (female, male)	Diseases's Condition	Comparisons	Outcomes	Nos
Yu (12)	2021	China	32	3.80, 1.73	23, 9	Tönnis:2,3,4	3D template vs. conventional	①②④⑤⑦	8
Zhou (13)	2022	China	31	NA	23, 8	Tönnis:2,3,4	3D + CAD VSS vs. conventional	①②④⑧	8
Pang (14)	2022	China	97	37.36, 8.5M	80, 17	Tönnis:3,4	3D template vs. conventional	①③⑤⑦	8
Zheng (15)	2017	China	25	10.66, 1.97	21, 4	Tönnis:2,3,4	Navigation template vs. conventional	①②④⑤⑥⑧	8
Cao (16)	2022	China	40	9.4, 2.1	12, 28	Tönnis:1,2,3,4	3D printing vs. conventional	①②③⑤	8
Shi (17)	2020	China	29	3.94, 1.92	22, 7	Tönnis:2,3,4	Navigation template vs. conventional	①②④⑥⑧	8
Chen (18)	2022	China	34	3.35, 1.04	21, 13	Tönnis:2,3,4	3D printing vs. conventional	①②③④⑤⑦	8

NOS, Newcastle–Ottawa-scale; NA, not reported. ① Duration of surgery. ② x-ray frequency during surgery. ③ Intraoperative bleeding. ④ Number of patients with McKay clinical function graded as poor. ⑤ Number of patients with Severin x-ray graded as poor. ⑥ Postoperative leg length discrepancy. ⑦ Postoperative acetabular index. ⑧ Proximal femur osteotomy angle.

### Results of meta-analysis

A total of 7 retrospective cohort studies were included. Publication bias assessment was unavailable due to less than 10 studies included. Sensitivity analysis was performed for duration of surgery, x-ray frequency during surgery, and intraoperative bleeding in that few studies reported relevant outcomes. The results were robust after removal of each study one by one, while significant heterogeneity remained (Table 2). Compared with conventional DDH osteotomy, 3D navigation-assisted osteotomy:

**Table 2:** The sensitivity analysis of the outcome.

Study	Mean	95%CI
**(A) X-ray frequency during surgery of sensitivity analysis**		
Omitting Cao 2022	−3.14	−3.93, −2.35
Omitting Chen 2022	−2.99	−3.82, −2.17
Omitting Shi 2020	−2.62	−3.02, −2.21
Omitting Yu 2021	−3.06	−3.77, −2.35
Omitting Zheng 2017	−3.02	−3.84, −2.20
Omitting Zhou 2022	−2.90	−3.63, −2.17
Total	−2.94	−3.58, −2.30
**(B) Duration of surgery of sensitivity analysis**		
Omitting Cao 2022	−21.53	−25.68, −17.39
Omitting Chen 2022	−21.36	−25.32, −17.19
Omitting Pang 2022	−22.8	−27.77, −17.82
Omitting Shi 2020	−24.01	−28.93, −19.08
Omitting Yu 2021	−23.97	−29.25, −18.70
Omitting Zheng 2017	−22.46	−27.31, −17.60
Omitting Zhou 2022	−24.01	−29.03, −18.99
Total	−22.86	−27.29, −18.43
**(C) Intraoperative bleeding of sensitivity analysis**		
Omitting Cao 2022	−35.09	−80.77, 10.60
Omitting Chen 2022	−17.08	−25.73, −8.43
Omitting Pang 2022	−39.3	−76.30, −2.30
Total	−26.83	−39.24, −14.41

#### Duration of surgery

**Figure 2:**
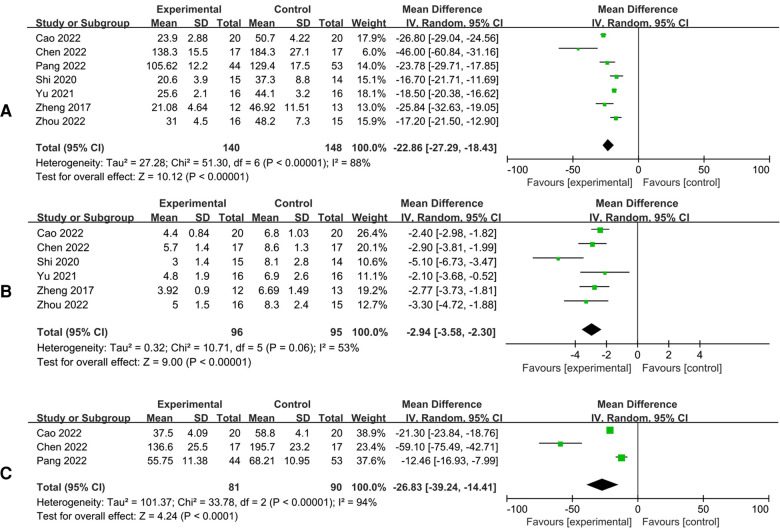
Forest plots of the overall meta-analysis. Operation time (**A**); Intraoperative fluoroscopy frequency (**B**). Intraoperative blood loss (**C**).

All included studies reported duration of surgery, with significant heterogeneity existing among the studies (*I*^2^*^ ^*= 88%), and random-effect model was applied for meta-analysis. The results showed that 3D navigation-assisted osteotomy remarkably decreased the duration of surgery [*I*^2^*^ ^*= 88%, REM, MD = 22.86, 95%CI (−27.29, −18.43), *p *< 0.00001] (Figure 2A).

#### x -ray frequency during surgery

There were 6 studies that reported x-ray frequency during surgery, and the results showed that 3D navigation-assisted osteotomy required lower x-ray frequency [*I*^2^*^ ^*= 53%, REM, MD = 2.76, 95%CI (−3.15, −2.37), *p *< 0.00001], while there was significant heterogeneity among the studies (*I*^2^*^ ^*= 53%) (Figure 2B).

#### Intraoperative bleeding

There were 3 studies that reported intraoperative bleeding volume, and the results showed that 3D navigation-assisted osteotomy had less intraoperative bleeding volume [*I*^2^*^ ^*= 94%, REM, MD = 26.83, 95%CI (−39.24, −14.41), *p *< 0.0001], while there was significant heterogeneity among the studies (*I*^2^*^ ^*= 94%) (Figure 2C).

#### Number of patients with McKay clinical function graded as poor

**Figure 3:**
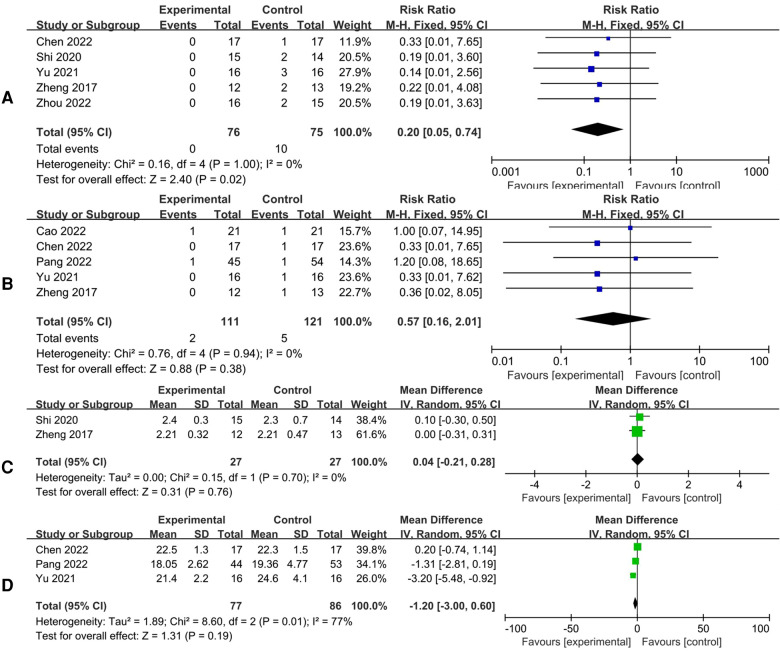
Forest plots of the overall meta-analysis. Poor results of McKay clinical function evaluation (**A**); poor results of severin X line assessment (**B**); postoperative lower limb discrepancy (**C**); postoperative acetabulum indx (**D**).

There were 5 studies that reported numbers of patients with McKay clinical function graded as poor. The results showed that patients receiving 3D navigation-assisted osteotomy has better clinical function [*I*^2^*^ ^*= 0%, FEM, RR = 0.20, 95%CI (0.05, 0.74), *p *= 0.02]. No significant heterogeneity considered existing among the studies (*I*^2^*^ ^*= 0%) (Figure 3A).

#### Number of patients with severin x-ray graded as poor

There were 5 studies that reported numbers of patients with Severin x-ray graded as poor. There was no significant difference between the two groups [*I*^2^*^ ^*= 0%, FEM, RR = 0.57, 95%CI (0.16, 2.01), *p *= 0.38]. No significant heterogeneity considered existing among the studies (*I*^2^*^ ^*= 0%) (Figure 3B).

#### Postoperative leg length discrepancy

There were 2 studies that reported postoperative leg length discrepancy. There was no significantly statistical difference between the two groups [*I*^2^*^ ^*= 0%, FEM, MD = 0.04, 95%CI (−0.21, 0.28), *p *= 0.76]. No significant heterogeneity considered existing among the studies (*I*^2^*^ ^*= 0%) (Figure 3C).

#### Postoperative acetabular index

There were 3 studies that reported postoperative acetabular index. There was no significant difference between 3D navigation-assisted osteotomy and conventional osteotomy [*I*^2^*^ ^*= 77%, REM, MD = −1.20, 95%CI (−3.00, 0.06), *p *= 0.03]. There was significant heterogeneity among the studies (*I*^2^*^ ^*= 77%) (Figure 3D).

#### Proximal femur osteotomy angle

There were 3 studies that reported postoperative proximal femur osteotomy angle in the two groups. These studies had assessed just 1 of the 3 proximal femur osteotomy angle-related dimensions, which included proximal femur varus, rotation, and shortening, so that this outcome could not be assessed.

## Discussion

This meta-analysis showed that 3D navigation-assisted hip osteotomy could be more effective than conventional hip osteotomy in the treatment of child DDH, and the difference was statistically significant. 3D navigation-assisted osteotomy presented more superior in shortening duration of surgery and reducing intraoperative x-ray frequency and bleeding than conventional osteotomy.

DDH children often have aberrant and complicated anatomical characteristics in their hip joints, bringing multiple difficulties to conventional hip osteotomy (19, 20). We compared the duration of surgery, intraoperative x-ray frequency, intraoperative bleeding, postoperative leg length discrepancy, postoperative acetabular index, number of patients with postoperative McKay clinical function (21) graded poor, and number of patients with Severin x-ray (22) graded as poor. The results revealed statistical significance in the duration of surgery, intraoperative x-ray frequency, intraoperative bleeding, postoperative acetabular index, and number of patients with postoperative McKay clinical function graded poor. There were no significantly statistical differences in the number of patients with Severin x-ray graded as poor and postoperative leg length discrepancy. However, it has been reported by several studies that there is a difference between preoperatively set osteotomy angles and postoperatively measured ones. We failed to further explore this difference due to few studies included. It can be determined that 3D navigation-assisted DDH hip osteotomy appears more advantaged in accuracy, compared with conventional osteotomy. The design of proximal femur osteotomy involves rotation, varus and shortening, which requires accurate osteotomy and proper placement of steel plate so as to achieve satisfied correction (23). Part of included studies reported the advantages of 3D navigation-assisted rotation, varus and shortening, which was also undiscussed in this study due to few studies included.

Most studies indicate that the older the children are, the more likely they are to need further surgical treatment for acetabular residual dysplasia (24–26). For DDH children aged over 2 years, osteotomy presents to be the most preferable option in that increased acetabular index and femoral neck anteversion are important pathological characteristics in DDH (27, 28). The goal of treatment is to achieve reduction of acetabular concentric circles so that to promote the recovery to normal anatomical structure and optimal joint function, which is also crucial for postoperative evaluation (29, 30). Pelvic osteotomy combined with proximal femur osteotomy can facilitate joint reduction, decrease stress on hip joint, correct over-anteversion, and reduce acetabular index, so that to finally restore the nearly normal anatomical structure around the hip joint (31, 32).

Conventional Salter pelvic rotational osteotomy can cause proximal and distal malposition. The supportive triangular bone block needs to be firmly fixed, and the fixation ends of the Kirschner wire should be kept contactless with hip joint, which demands accurate fixation direction (33). Child often has thin ilium, and attempts to fix could induce unfirm fixation and subsequent bone block shift or collapse so that the therapeutic effect would be compromised. Generally, this process is performed by experienced clinicians. Aberrant anatomical characteristics of the hip joint in DDH children (34) increase the difficulty and risk of pelvic and femur osteotomy (35). Conventional osteotomy scheme is made typically based on preoperative pelvic x-ray scan and computed tomography (CT), which is less intuitive so that the osteotomy requires a wealth of experience and exquisite skills of the surgeon.

The pelvis and femur models were reconstructed in three dimensions by importing the original DICOM data of the hip joint into professional software. According to preoperative measurements and compared with the contralateral parameters, design the size of the pelvic bone cutting way, direction, internal fixation, and varus femoral Angle, rotation Angle and length of bone cutting, with reference to the intraoperative use of steel plate pitch, gram parameters such as diameter of needle, the needle was simulated bone cutting plane and into a certain way, navigation template design and the reverse calculation combined with modeling, The surface anatomical and morphological features of pelvis and femur were extracted, and the navigation template three-dimensional model with Kirschner needle path was established. A 1 : 1 hip joint model can be constructed through 3D navigation, which allows orthopedic surgeons to simulate the whole surgical process, make full assessment of the key points during surgery, and select optimal surgical option and scheme. On the other hand, pelvic and femur osteotomy and internal fixation *via* navigation template can prevent shifting or over-deep implantation to ensure accurate osteotomy and internal fixation. Inexperienced surgeon can also perform the osteotomy with the aid of 3D navigation. The learning curve is shortened (36). In addition, orthopedic surgeons can conduct preoperative communication with patients using 3D printed model, which brings the patients more intuitive understanding of the surgical procedure, enhances their confidence, and increases their satisfaction (13).

A study by Liu et al. (16), found that application of 3D printed pelvic model could increase accuracy and shorten duration of the surgery so that increase the success rate of osteotomy in DDH children and promote their postoperative recovery. Therefore, 3D navigation-assisted pelvic osteotomy can manifest the pelvic deformity of DDH child more intuitively *via* individualized surgical navigation template so as to help surgeons make accurate measurements, perform surgery simulation, make individualized scheme. It can significantly increase accuracy of the surgery, reduce intraoperative bleeding and radiation exposure, and shorten duration of the surgery. Acetabular index is the most reliable imaging indicator to evaluate the development of DDH (12). This study found that DDH patients receiving 3D navigation-assisted osteotomy had significantly lower postoperative acetabular index than those receiving conventional osteotomy.

Application of 3D navigation promotes the development of orthopedic surgery, while it also needs further improvement (17). The increased surgery costs may restrict the popularization and application of this technology (14, 15). Additionally, the 3D printing equipment is too expensive and the skill requirement is too complicated to be afforded by many hospitals (18). The surgical scheme-making by 3D navigation requires long enough time so that it may be only applicable for patients receiving selective surgery. The contribution of 3D navigation to the decrease of postoperative complications has yet been revealed due to few relevant studies. Meta-analysis based on more clinical trials is needed to robustly support its application.

## Limitations

The results of this meta-analysis are reliable in that the study selection is based on strict inclusion criteria, and the conclusion is drawn *via* standard meta-analysis process. However, several limitations do exist: firstly, few studies have included, and some of them had limited sample size, resulting in bias of the results. Reliability of the conclusion should be further validated; Secondly, multiple factors could affect the performance of DDH hip osteotomy. Although heterogeneity across sensitivity analyses was stable across all analyses, the large heterogeneity still calls for caution in interpreting the results. The source of heterogeneity may be related to the few included literatures and sample size, different included population, different age, and different application methods of 3D navigation technology. More randomized and large-sample clinical studies are needed in the future. The reviewers have conduct strict screening and selection during data extraction but have failed to perform subgroup analysis. More detailed data should be collected for follow-up studies.

## Conclusion

3D navigation-assisted pelvis and thighbone osteotomy for DDH children could shorten duration of surgery and reduce intraoperative bleeding and x-ray exposure, presenting definite therapeutic effect. As for postoperative outcomes, 3D navigation-assisted hip osteotomy is demonstrated to be more effective than conventional osteotomy. The conclusion of this meta-analysis needs to be further validated and updated by more RCTs with remarkable quality. Expanding application of 3D navigation-assisted hip osteotomy presents a great trend in future orthopedic surgery.

## Data Availability

The original contributions presented in the study are included in the article/Supplementary Material, further inquiries can be directed to the corresponding author/s.
